# Supplementary material for: Trend of HPV Molecular Epidemiology in the Post-Vaccine Era: A 10-Year Study
Source: Viruses. 2023 Sep 27;15(10):2015. doi: 10.3390/v15102015 (PMC10612033; doi:10.3390/v15102015)
Supplement: Supplementary file 1 [file viruses-15-02015-s001.zip › viruses-2597081-supplementary.pdf]

Table S1. Trend change of high risk and non-high risk HPV genotype infections over time in different age groups.

Test trend for different age group

Age group	High risk	Non high risk
>70	0.7561	0.3262
20-30	0.9583	0.9962
30-40	0.3546	0.7012
40-50	0.1203	0.0022
50-60	0.4213	0.0036
60-70	< 0.001	< 0.001
0-20	1.000	0.8276

Table S2. Statistical testing of high risk and non-high risk genotypes under different age groups over 2010-2020.

Comparisons of high risk and non high risk each year under each age group

	Age group						
**Year**	**>70**	**20-30**	**30-40**	**40-50**	**50-60**	**60-70**	**0-20**
**2010**	0.4901	0.2193	0.0022	0.0008	0.0878	0.1396	NA
**2011**	0.9858	0.7197	0.0323	0.2720	0.0035	0.2383	NA
**2012**	0.0610	0.6448	0.0001	1	0.0001	0.1194	0.1463
**2013**	0.6565	1	0.0019	0.4942	0.0150	0.9130	0.4667
**2014**	0.5543	0.0501	0.0004	0.5176	< 0.0001	0.3573	NA
**2015**	0.6480	0.2421	< 0.0001	0.2729	0.0002	0.0099	0.4576
**2016**	0.5099	0.0275	0.0006	0.7809	0.1109	0.0001	0.4657
**2017**	0.6859	0.5246	0.0003	0.2586	0.0152	0.3353	NA
**2018**	0.4952	0.0179	0.0003	0.1523	0.6429	0.0026	0.2143
**2019**	0.7299	0.6252	< 0.0001	0.7256	0.0201	0.0538	1
**2020**	0.4838	0.9661	0.2692	0.2533	0.0256	0.9611	NA

Table S3. Statistical testing of trend change for the HPV genotypes.

Genotype	p-value
HPV-16	0.6824
HPV-18	0.6335
HPV-31	0.018
HPV-33	0.0089
HPV-35	0.0901
HPV-39	<0.001
HPV-45	0.8749
HPV-51	<0.001
HPV-52	0.0972
HPV-56	0.5243
HPV-58	0.0075
HPV-59	<0.001
